# Principal goals at school: evaluating construct validity and response scaling format

**DOI:** 10.3389/fpsyg.2023.1283686

**Published:** 2024-01-23

**Authors:** Faye Antoniou, Mohammed H. Alghamdi

**Affiliations:** ^1^Department of Educational Studies, National and Kapodistrian University of Athens, Athens, Greece; ^2^Department of Self-Development Skills, King Saud University, Riyadh, Saudi Arabia

**Keywords:** principal goals, SASS survey, response scaling, item response theory, collapsing rating scale categories

## Abstract

The purpose of the present study was to test the efficacy and appropriateness of the 4-point response option of the Principal’s Goals Scale of the SASS (1999–2000) survey. Competing dichotomous models with various conceptualizations were constructed and tested against the original polytomous conceptualization. Participants were 8,524 principals from whom 64% were males and 36% females. Principals’ goals were assessed using a 6-item scale anchored across points reflecting proximity to achieving a goal. The original polytomous conceptualization was contrasted to a dichotomous two-pole conceptualization using a model with freely estimated discriminations (two-parameter logistic model, 2PL) as well as the Rasch model assuming equal discrimination parameters. Results indicated that the 2PL dichotomous model provided the most optimal model fit. Furthermore, item-related, and person-related estimates pointed to enhanced accuracy and validity for the dichotomous model conceptualization compared to the polytomous model. It is suggested that a dichotomous scaling system is considered in subsequent measurements of the scale as a means of enhancing the accuracy and validity of the measured trait.

## 1 Introduction

Principals of schools should make it a priority to develop ambitious objectives for their institutions since doing so may have a beneficial effect on many facets of the learning environment and the results for students. Creating a culture in which high expectations are the norm among instructors, students, and parents may be accomplished by setting lofty objectives. According to research conducted by Jussim and Harber (2005), having high expectations from instructors has a beneficial effect on the academic performance of their students. When administrators set lofty objectives for their schools, they inspire every member of the school community to strive for greatness, which in turn leads to an increase in both effort and engagement. In addition, research has indicated that schools with strong leadership and clear objectives tend to have greater levels of student accomplishment (Hallinger and Heck, 1996; Leithwood et al., 2004). There is a correlation between principals who have high standards for academic success and those who provide an atmosphere of support for both teachers and students (Bozkurt, 2023; Perkasa et al., 2023). This correlation contributes to enhanced learning outcomes.

To boost teacher retention rates and promote professional development programs, setting ambitious targets may be quite helpful. The development of a feeling of professional progress and happiness in one’s work is facilitated when administrators articulate an aspiration for academic superiority and provide teachers with the resources necessary to realize that aspiration. According to Hanushek et al. (2004), this, in turn, serves to contribute to the overall quality of education and helps the school retain excellent educators inside the institution. Furthermore, ambitious objectives inspire principals and their teams to seek out creative techniques and apply evidence-based solutions. According to Leithwood et al. (2008), principals can create positive changes in teaching techniques, curriculum design, and school-wide. Policy by cultivating a culture of continuous improvement in their schools. This ultimately results in improved educational experiences for students. According to several studies, one of the ways in which administrators may realize their lofty objectives is by incorporating the stakeholders in the process, as well as the school community, the parents, and the organizations in the surrounding area. According to Epstein (2001), stakeholders, in particular, have the potential to provide resources, opportunities, and enriched experiences, as well as further help in the development of a productive and collaborative atmosphere that contributes to students’ overall well-being and academic performance.

At present, several national and international studies have investigated the role and functioning of principals as well as the consequences of their actions. One such study is the “School and Staffing Survey” which mainly collects information from principals regarding school functioning, their roles, and responsibilities as well as their perceived obstacles and barriers to achieving their goals. In the present study, we focus on the principal’s goals as we target at re-examining the psychometric qualities of the specific instrument. Besides reliability and construct validity, we are additionally interested in the response scaling system employed as it deviates markedly from Likert-type or frequentist systems. Thus, what is least known, is the efficacy of the response scaling format as the current 4-point scaling system could be suboptimal compared to other available systems, e.g., a dichotomous conceptualization. Currently, scoring includes summed responses of the original 4-point scaling system and validity studies have utilized the total score as a means of estimating total scores. If, however, the current scaling response option proves to be suboptimal, then the associated total scores will have to be revised accordingly in subsequent international measurements.

The literature on survey methods (e.g., Tourangeau et al., 2000) suggests that there are at least three salient contributing factors to consider revising a scale system namely, alignment with other measures, infrequent use of some rating scale options, and conceptual redundancy (Rutkowski et al., 2019). The first refers to harmonizing the scale’s definition with those of other instruments that are valid or are considered gold standards. Harmonizing answer categories becomes important when researchers want to compare their findings to those of prior studies or make links between other dimensions (Dusen and Nissen, 2020). Researchers may compare and more easily integrate their results by ensuring uniformity and compatibility across studies by compressing answer possibilities. The second reason for collapsing categories refers to when certain choices are infrequently used (Groves et al., 2011). In many situations, collapsing facilitates data analysis by minimizing the number of categories and enhancing interpretability and statistical power. Response choices that are rarely chosen may not provide useful data or may impede analyses by leaving blank cells or sparse categories (Krosnick and Fabrigar, 1997; Agresti, 2013) as is the case with the omnibus chi-square test that evaluates global model fit. The third refers to the phenomenon when adjacent categories are conceptually similar to the extent that their differentiation is neither clearly defined nor easily attained, thus threatening the reliability of measurement (Embretson and Reise, 2000). On the other hand, the disadvantages of collapsing categories in a rating scale have been a reduction of power (Strömberg, 1996), problems with model convergence (Savalei and Rhemtulla, 2013), distorted model fit (Jeong and Lee, 2016), and loss of reliability and information (Embretson and Reise, 2000; Revilla et al., 2017). Applications of revising scale systems have utilized the constructs of bullying (Rutkowski et al., 2019), personality (Wetzel and Carstensen, 2014), disability status (Dadaş et al., 2020), academic misconduct (Royal et al., 2015), and health status (Williams et al., 2009). The purpose of the present study was to test the efficacy and appropriateness of the 4-point response option of the Principal’s Goals Scale of the SASS survey. Competing dichotomous models with various conceptualizations were constructed and tested against the original polytomous conceptualization.

## 2 Methods

### 2.1 Participants and procedures

Participants were 8,524 principals who participated in the School and Staffing Survey during 1999–2000. There were 5,481 males (64.3%) and 3,043 females (35.7%). Most principals were above 50 years old (53.7%). There were 348 Hispanic principals representing 4.1% of the total sample. Regarding race, 87.1% were white followed by black (9.9%), American Indian (1.8%), and Asian (1.2%). All but 1.6% had at least a Master’s degree. The sampling frame in SASS used the Common Core of Data (CCD) file that includes all elementary and secondary schools in the USA. Sampling in the SASS involved school selection using a probability proportionate to the square root of the number of teachers. Data collection was performed by the U.S. Census Bureau using advance and follow-up letters to the schools and the mode of data collection was computer-assisted telephone interviewing.

### 2.2 Measure

The principal’s goals scale (see Appendix 1) is a six-item scale anchored between a 4-point scaling format ranging from a goal that is far or close to being reached. The potential nominal type scaling with ordered but likely non-equidistant options was a primary motivating factor for evaluating the instrument’s response scaling system. Furthermore, scale selection was based on utility as there were 190 published papers or presentations using the specific instrument, with reports confirming adequate levels of reliability and validity (e.g., Blank, 1994).

### 2.3 Data analyses

#### 2.3.1 Construct validity and person consistency

Data were analyzed using Item Response Theory (IRT) and by employing the Graded Response Model (Samejima, 1969; Muraki, 1992) which is appropriate for polytomous data and a series of models for dichotomous items, namely the Rasch model and the 2-parameter IRT model (2PL). Besides the polytomous model, a dichotomy of the 4-scaling system format was created by aggregating the two positive against the two negative responses. Model fit was evaluated using the omnibus chi-square test and the Root Mean Square Error of Approximation (RMSEA). Further local tests included item misfits using chi-square tests and tests of local dependency using the LD index. Given the polytomous nature of the original scaling system, another means of examining scaling appropriateness was the equidistant index (Spratto, 2018), which evaluates the difference between adjacent thresholds, assuming equal distances between rating scale options. Given that thresholds are evaluated in logits, the expected value of the null hypothesis of no differences is equal to zero logits. Although the scaling system deviates markedly from other Likert-type conceptualizations, it was important to examine whether the conceptual distance between “just beginning” and “long way to go,” assuming this is the low goal attainment pole, was equivalent to the distance between the “almost there” and “reached our goal” options. Threshold non-equivalence would have implications for psychometrics as the scaling would no longer be considered on the interval scale but should be viewed either as ordered data or even at the nominal level.

Further tests for determining the appropriateness of the scaling system involved the examination of 108 person location fluctuations around the latent trait, termed Person Discriminal Dispersion (PDD) (Ferrando, 2007, 2009; Ferrando and Navarro-González, 2021) which refers to the consistency of the response patterns of individuals about variable item locations (Ferrando, 2016). Well-fitted participants have low values in their discriminal dispersion showing enhanced consistency (Ferrando, 2019). Ferrando and Navarro-González (2020) developed the R package InDisc to provide sample-based estimates of both global fit and person dispersion estimates (R Core Team, 2018). In the present study, we contrasted average estimates of person dispersion between polytomous and dichotomous conceptualizations as a means of evaluating the consistency of the person trait estimates.

#### 2.3.2 Internal consistency reliability

It was assessed using Marginal reliability in light of the recommendations disfavoring Cronbach’s alpha as being a low-bound estimate (Sijtsma and Molenaar, 1987; Sijtsma, 2009). Estimates were 0.76 for the polytomous model 0.56 for the 2PL dichotomous conceptualization and 0.45 for the dichotomous conceptualization with fixed slopes (Rasch model).

## 3 Results

### 3.1 Model fit as a function of different response scaling formats

**Figure 1:**
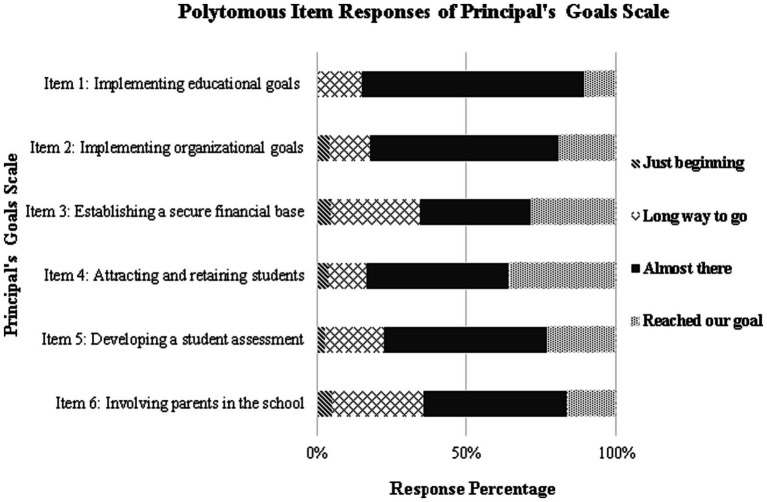
Frequency of response categories of the principal’s goal scale.

A Graded Item Response model was fit to the data as per the original conceptualization. As shown in Table 1, the omnibus chi-square test was significant but unstandardized residuals (i.e., RMSEA) were within the normal range (i.e., 3%). A visual analysis of the items’ category curves, however, showed substantial underrepresentation of the “just beginning” category suggesting it was not by itself constructive for measurement purposes (see Figure 1). This finding had significant implications for rating scale equivalence. As shown in Table 2, the conceptual non-equivalence between adjacent thresholds was confirmed as the two poles occupied significantly different spaces across theta. On items 1, 2, 4, and 5, the positive sign of the equidistance index suggests that the distance in thresholds 2 and 3 is significantly larger compared to that of thresholds 1 and 2. Thus, the threshold non-equivalence testing provided some evidence of the lack of optimal functionality of the scaling system.

**Table 1:** Model fit for principal’s goals scale using polytomous and dichotomous models.

Model tested	Chi-square	D.F.	value of *p*	RMSEA	AIC	BIC	Omega
M1. Polytomous Graded	26674.39***	4,071	<0.001	0.03	95641.16	95810.37	0.738
M2. Dichotomous-2PL	197.84***	51	<0.001	**0.02**	**47486.52**	**47571.13**	0.652
M3. Dichotomous Rasch	1186.02***	57	<0.001	0.05	48447.57	48489.88	0.453

D.F., Degrees of freedom; RMSEA, Root Mean Squared Error of Approximation; AIC, Akaike Information Criterion; BIC, Bayesian Information Criterion; Omega, index of internal consistency reliability. Bold values indicate optimal model with the smallest values in the information criteria (AIC, BIC), the RMSEA and the chi-square statistic. ***p < 0.001.

**Table 2:** Equidistance between thresholds in the principal’s goals scale.

Item	Discrimination	Threshold/S.E.		Difference/S.E. of Diff		*Z*-test	Equidistance index
Item 1	2.368	−5.270	0.120	–	–	–	–
		−2.639	0.069	1.111	0.034	-	-
		3.726	0.088	2.688	0.042	34.382***	1.577
Item 2	1.821	−4.417	0.086	–	–	–	–
		−2.274	0.051	1.177	0.038	–	–
		2.167	0.049	2.439	0.044	26.379***	1.262
Item 3	0.929	−3.594	0.065	–	–	–	–
		−0.786	0.028	3.023	0.109	–	–
		1.063	0.030	1.990	0.063	12.545***	−1.032
Item 4	1.467	−4.665	0.098	–	–	–	–
		−2.236	0.048	1.656	0.066	–	–
		0.815	0.034	2.080	0.050	6.446***	0.424
Item 5	1.385	−3.780	0.067	–	–	–	–
		−1.504	0.036	1.643	0.051	–	–
		1.652	0.037	2.279	0.049	12.362***	0.635
Item 6	1.327	−3.969	0.071	–	–	–	–
		−0.817	0.031	2.375	0.066	–	–
		2.099	0.041	2.197	0.050	3.06**	−0.178

The equidistance index evaluates the difference between pairs of adjacent thresholds (i.e., 1 and 2 vs. 2 and 3). ***p < 0.001; **p < 0.01.

In light of the above findings on omnibus model fit and threshold non-equivalence, the two adjacent content categories “just beginning” and “long way to go” were aggregated to define the first level of a dichotomy (i.e., zero) with the categories “almost there” and “reached our goal” representing the next category (i.e., one). As shown in Table 1, the smallest chi-square value was reserved by the 2PL model, although the chi-square estimate was significant signaling the expected excessive levels of power. Unstandardized residuals were 2% suggesting “exact model fit” as per MacCallum et al. (1996) recommendations. The second-best model was the dichotomous Rasch model with, however, a significant misfit over the 2PL model by freeing the estimation of 6 discrimination parameters. Given that models were nested, a chi-square difference test pointed to the superiority of the dichotomous 2PL model compared to the Rasch model [∆*_Chi − square_* (6) = 988.180, *p* < 0.001]. In other words, fixing the discrimination parameters to unity was associated with 988 units of model misfit. The polytomous-graded model was by far the worst estimated model. Noteworthy, RMSEA was still acceptable. Thus, global statistical criteria favored a dichotomous response option with two poles as being the most parsimonious and errorless conceptualization for the measurement of principals’ attitudes toward their school’s goals.

### 3.2 Item fit statistics: response patterns and residual correlations

Tests of local dependency showed non-significant residual correlations for only the dichotomous 2PL model (see Table 3). Both the dichotomous Rasch model and the polytomous model were associated with significant residual correlations; for the polytomous model, the residual correlations were extended to all pairs of items. For the dichotomous Rasch model, residual correlations were significant across all pairs except items 1 and 3; items 3 and 4; and items 3 and 5. Residual correlations represent a significant obstacle to the validity of person scores as they violate an important prerequisite assumption of the IRT modeling. Furthermore, the fact that two items correlate with each other at the level of variance not explained by the latent construct is both problematic and creates interpretation issues. Thus, collectively, all the evidence pointed to the superiority of the dichotomous 2PL model over the original polytomous model as a more parsimonious and valid assessment of the principal’s goals at school.

**Table 3:** Between item residual correlations for principal’s goals scale across tested models.

Principal Scale Items	Item 1	Item 2	Item 3	Item 4	Item 5	*χ*^2^/D.F.
Polytomous data-graded model						
Item 1: implementing educational goals	–	–	–	–	–	123.27***/32
Item 2: implementing organizational goals	68.50***	–	–	–	–	99.93***/35
Item 3: establishing a secure financial base	27.40**	35.80***	–	–	–	200.97***/41
Item 4: attracting and retaining students	47.70**	38.20**	68.50***	–		170.85***/39
Item 5: developing a student assessment	34.80***	44.70**	25.40**	24.00**	-	86.38***/37
Item 6: involving parents in the school	36.50**	42.40**	22.00**	38.20***	40.10***	103.32***/38
Dichotomous data-rasch model						
Item 1: implementing educational goals	–	–	–	–	–	356.39***/5
Item 2: implementing organizational goals	483.20***		–	–	–	200.79***/5
Item 3: establishing a secure financial base	0.30	7.40*	–	–	–	45.78***/5
Item 4: attracting and retaining students	138.40***	70.30***	39.80***	–	–	127.04***/5
Item 5: developing a student assessment	178.40***	92.40***	6.20	28.80***	–	48.90***/5
Item 6: involving parents in the school	131.10***	44.60***	1.80	65.00***	40.60***	46.40***/5
Dichotomous data-2PL						
Item 1: implementing educational goals	–	–	–	–	–	19.39**/4
Item 2: implementing organizational goals	1.80		–	–	–	17.77**/4
Item 3: establishing a secure financial base	7.00	0.00	–	–	–	29.11**/4
Item 4: attracting and retaining students	3.20	2.80	37.30***	–	–	26.71**/4
Item 5: developing a student assessment	−0.70	−0.30	2.70	0.80	–	21.83**/4
Item 6: involving parents in the school	−0.10	5.60	−0.60	7.00	1.10	21.21**/4

***p < 0.001; **p < 0.01; *p < 0.01. Non-significant values are indicative of good model fit.

Further tests of local model fit (i.e., at the item level) utilized the chi-square test to evaluate discrepancies between observed and expected response patterns. As shown in Table 3, the only model for which items fitted the data properly was the dichotomous, 2PL model; both the polytomous and the dichotomous Rasch conceptualization were associated with significantly elevated misfit as evidenced by the very large chi-square values.

### 3.3 Contrasting rating scale systems using person-based consistency in theta

As described above, estimates of person consistency on latent scores were evaluated in the different scaling systems using the R package InDisc (Ferrando and Navarro-González, 2020). The package provides estimates of global fit such as chi-square statistics and descriptive fit indices such as the Tucker Lewis Index (TLI) and RMSEA. Furthermore, average PDD values are also estimated. When contrasting polytomous versus dichotomous conceptualizations, results indicated a good model fit for only the dichotomous conceptualization [Chi-square(9) = 212.16, TLI = 0.935, RMSEA = 0.056] but not the polytomous one [Chi-square(9) = 442.46, TLI = 0.896, RMSEA = 0.109]. More so, average indices of personal dispersion were 0.51 for the dichotomous conceptualization and 0.66 for the polytomous one. Knowing that lower values are indicative of higher consistency, the dichotomous 2PL model is most likely preferred over the polytomous model.

## 4 Discussion

The purpose of the present study was to test the efficacy and appropriateness of the 4-point response option of the Principal’s Goals Scale of the SASS survey, with the additional goal of proposing the testing rather than implied psychometric properties of instruments and specifically the functioning of their rating scale. Competing dichotomous models with various conceptualizations were constructed and tested against the original polytomous conceptualization.

The present study found that the originally formed scaling system of the principal’s goal scale was not associated with an optimal model fit and measurement precision using both scale-level and person-level criteria. Two alternative dichotomous scaling systems were tested, one with free and one with fixed (Rasch) discrimination parameters with the freely estimated 2PL model being associated with the most optimal model fit. The evidence overwhelmingly favored the dichotomous 2PL model as evidenced using fewer numbers of Guttman-related errors using the person dispersion estimates, enhanced amounts of information, and significantly improved model fit. The number of response categories for self-reports of pain interference was investigated in a study by Cook et al. (2010) which found that fewer response categories, as few as five or six, may function as well as the 11 response categories that are conventionally used. However, the results are preliminary since the number of response categories presented was not manipulated in the study design. Therefore, future research should compare the reliability and validity of scores based on the original number of response categories versus a presentation with fewer response options. When scoring assessments, Dusen and Nissen (2020) advised sparingly using data manipulations and keeping all answer categories unless there was a compelling reason to collapse them. They spoke about how to experimentally test for the two probable causes of falling answer categories: loss of utilization and redundancy.

The difficult process of updating a scale system illustrates the scientific community’s ongoing efforts to accurately reflect and quantify psychological factors. This project requires balancing dependability and validity. Empirical research relies on reliability and validity, the foundations of robust measurement. Redefining clinical levels by adjusting cutoff values and threshold estimates shows how theory and measurement interact. Modifying scaling systems, however difficult as a task, likely improves construct validity. This improves the accuracy of score-based inferences and conclusions. For example, a depression or anxiety scale may revise its scaling system to redefine the clinical levels of these constructs. The process of revising scaling systems in this particular context functions as a means to recalibrate the operational concepts that form the foundation of these constructions. Its significance cannot be overstated since these latent constructs need to account for the fluctuations of the diagnostic criteria as they are oftentimes altered as they are informed by new empirical findings.

### 4.1 Study limitations

The present study is limited for several reasons. First, the use of PDDS is rather new and reflects a rather underexplored aspect of person fit. As Ferrando and Navarro-González (2020) stated, the sensitivity of PDD estimates is a function of test length with larger tests having enhanced confidence in the stability of the estimated parameters. Second, models were likely overpowered with *n* = 8,500 participants, thus, global fit statistics are likely inflated for these reasons leading to rejections of model fit, even when discrepancies between hypothesized and observed models are not large. The data used pertain to a national database and an instrument that was mostly used between 1999 and 2010, thus, later inferences about the instrument cannot be made. Last, the comparisons between models should take into account the fact that models with fewer categories are artificially inflated for the better as items become more similar, thus, models cannot be contrasted in terms of, e.g., precision as such a finding is attributed to collapsing categories.

### 4.2 Conclusions and future directions

Before a choice can be taken, the finding that nearby categories need to be merged into a single category has to be verified using other data sets. Researchers must evaluate the final scaling system to ensure that it is relevant, accurately represents the data, and does not compromise the content validity of the study. A collapsing based only on statistical criteria alone is not suggested since low frequency should not be the basis for collapsing; certain significant and useful metrics have a low frequency in the population, but this should not be the main reason for collapsing.

## Data availability statement

Publicly available datasets were analyzed in this study. This data can be found at: https://nces.ed.gov/surveys/sass/question9900.asp.

## Ethics statement

The studies involving humans were approved by all this was arranged by NCES at: https://nces.ed.gov/surveys/sass/question9900.asp. The studies were conducted in accordance with the local legislation and institutional requirements. The participants provided their written informed consent to participate in this study.

## Author contributions

FA: Conceptualization, Formal analysis, Investigation, Methodology, Visualization, Writing – original draft, Writing – review & editing. MA: Formal analysis, Funding acquisition, Investigation, Methodology, Project administration, Software, Supervision, Writing – review & editing, Writing – original draft.

## Appendix

SASS scale on principal’s goals for their school.

**Table:**
